# Recombinant factor IX Fc prophylaxis reduces pain and increases levels of physical activity, with sustained, long-term improvements in patients with hemophilia B: *post hoc* analysis of phase III trials using patient-reported outcomes

**DOI:** 10.1177/20406207231170701

**Published:** 2023-05-29

**Authors:** Jan Astermark, Cédric Hermans, Monia Ezzalfani, Alaeddine Sidhom, Sylvaine Barbier, Nana Kragh, Aletta Falk, Daniel Eriksson

**Affiliations:** Department of Translational Medicine, Lund University, JanWaldenströms gata 14, SE-205 02 Malmö, Sweden; Department of Haematology, Oncology and Radiation Physics, Skåne University Hospital, Malmö, Sweden; Department of Haematology, Oncology and Radiation Physics, Skåne University Hospital, Malmö, Sweden; Division of Haematology, Haemostasis and Thrombosis Unit, Saint-Luc University Hospital, Université Catholique de Louvain (UCLouvain), Brussels, Belgium; Putnam PHMR, Les Berges du lac, Tunisia; Putnam PHMR, Les Berges du lac, Tunisia; Putnam PHMR, Lyon, France; Swedish Orphan Biovitrum AB, Stockholm, Sweden; Swedish Orphan Biovitrum AB, Stockholm, Sweden; Swedish Orphan Biovitrum AB, Stockholm, Sweden

**Keywords:** factor IX, hemophilia B, pain, patient-reported outcome measures, physical activity, quality of life, rFIXFc protein

## Abstract

**Background:**

Pain is a common symptom of hemophilia that may adversely affect patients’ quality of life (QoL). Previous *post hoc* analyses of prophylaxis with recombinant factor IX Fc fusion protein (rFIXFc) have been published for adults and adolescents, demonstrating improvements in health-related QoL (HRQoL) when assessed by the haemophilia-specific QoL (HaemAQoL) questionnaire.

**Objective:**

To describe in depth the evolution of QoL, pain- and activity-related domains and questions for pediatric, adolescent, and adult patients with hemophilia B treated with rFIXFc prophylaxis.

**Design:**

A *post hoc* analysis of data from a series of clinical trials.

**Methods:**

This *post hoc*, long-term analysis assessed patient-reported outcomes (PROs) from the Kids B-LONG (NCT01440946: pediatric) and B-LONG (NCT01027364: adults and adolescents) parent studies and the B-YOND (NCT01425723: all age groups) extension study.

**Results:**

Ninety-two adult and adolescent patients that started in the B-LONG study were assessed, with a median (range) duration of follow-up of 58.9 (0.0–78.4) months. The Haem-A-QoL total score was significantly reduced from baseline by 4.45 (*p* ⩽ 0.01), as were the subdomains ‘physical health’ (9.10; *p* = 0.001), ‘sports and leisure’ (11.25; *p* ⩽ 0.01), ‘treatment’ (2.69; *p* = 0.05), and ‘view of self’ (5.81; *p* = 0.002). Thirty pediatric patients that started in the Kids B-LONG study were assessed, with a median (min–max) duration of follow-up of 36.7 (9.0–59.9) months. The high level of satisfaction demonstrated by the PROs at baseline was maintained.

**Conclusion:**

rFIXFc prophylaxis reduced perceived pain and increased levels of physical activity with sustained, long-term improvements in QoL in adult and adolescent patients with hemophilia B and maintained high QoL scores in pediatric patients.

## Plain language summary

Helping to reduce pain and increase physical activity in patients with hemophilia B

People with hemophilia B do not produce factor IX (FIX) that works properly, so they need to be given additional FIX to help their blood clot. Recombinant factor IX Fc fusion protein (rFIXFc), is an extended half-life (meaning it remains active for longer than standard, unmodified FIX) treatment for hemophilia B. People with hemophilia B can experience episodes of bleeding, which can result in other symptoms, including pain, difficulty participating in sport, and poor mental health. This study shows that regularly taking rFIXFc over approximately 5 years to prevent or treat bleeds could also help to make these other symptoms better.

## Introduction

Repeated bleeding episodes, along with prolonged bleeding after injury, are the hallmarks of hemophilia B.^1^ The majority of clinical bleeding episodes occur in the large mobile joints, and these hemarthroses can progress to synovial hypertrophy and irreversible joint damage.^2^ Hemarthrosis typically presents as acute pain and inflammation, due to distension of the joint capsule and functional disability, with repeated subacute episodes leading to hemophilic arthropathy with persistent pain in the joint during physical activity and, in some cases, at rest.^2^

While enduring and debilitating pain is well documented in patients with hemophilia,^3^ it is possible that coping strategies adopted by patients to deal with chronic pain may mask the full extent of the physical and mental burden.^4^ Furthermore, it has been shown that patients with severe hemophilia have poorer health-related quality of life (HRQoL) compared with the reference population.^2^ Measuring pain and quality of life (QoL) is subjective and personal,^4^ with patients describing hemophilia-associated pain as ‘intolerable, unbelievable and like being shot’.^3^ Patient-reported outcome (PRO) measures, such as Haemophilia Quality of Life Questionnaire for Adults (Haem-A-QoL), European Quality of Life Five Dimension (EQ-5D-3L), the Hemophilia-specific Treatment Satisfaction Questionnaire (HEMO-SAT), and the Canadian Haemophilia Outcomes–Kids’ Life Assessment Tool (CHO-KLAT), can help to quantify treatment benefit from the patient perspective.^5^

While physical activity is endorsed for patients with hemophilia,^5^ overall activity levels are lower than the healthy control population,^6^ despite advances in treatment allowing more patients to participate in physical activities.^5^ Indeed hemarthrosis is reduced and HRQoL improved by regular prophylaxis, regardless of age.^7^ Physical activity has the potential to improve pain and health status, reduce the risk of bleeding, and improve mental health.^8–11^ Therefore, early intervention is needed to prevent bleeding, promote physical activity, and improve general health.^12^ In support of this, prophylaxis initiated early in life has been associated with a reduction in bleeding and promotes psychosocial wellbeing and QoL.^5^ Moreover, a study of patients who did not initiate prophylaxis, at or before the age of 3, perceived that mental and physical health was poorer than the reference population.^13^ The current treatment goal is to relieve both physical and mental burden, resulting in living with a ‘hemophilia-free mind’.^14^

Recombinant factor IX Fc fusion protein (rFIXFc) is an extended half-life (EHL) factor IX (FIX) replacement therapy approved for the on-demand and prophylactic treatment of patients of all ages with hemophilia B.^15,16^ The safety and efficacy of rFIXFc was demonstrated in three phase III trials of previously treated pediatric (<12 years; Kids B-LONG)^17^ and adolescent/adult patients (⩾12 years; B-LONG)^18^ with severe hemophilia B (defined as endogenous FIX ⩽2 IU/dl), with patients completing the studies eligible to participate in the B-YOND long-term extension study.^19^ The long-term safety and efficacy of rFIXFc was confirmed when patients from Kids B-LONG and B-LONG were followed, on average, for 4 or 5 years, respectively, in the extension trial (cumulative duration up to 6.5 years in adults and adolescents, and 4.8 years in children; B-YOND).^19^

*Post hoc* analyses using data from the B-LONG trial for adults and adolescents highlighted improvements in both pain and physical activity (as measured by the EQ-5D-3L domains of ‘Pain/Discomfort’ and ‘Mobility’, respectively) irrespective of their treatment regimen prior to receiving rFIXFc.^20^ Results from Haem-A-QoL demonstrated that patients reported meaningful improvements in ‘Total score’, and the subdomains ‘Physical health’ and ‘Sports and leisure’,^21^ which were maintained over 24 months in an interim assessment of BYOND.^22^

This study presents a *post hoc* analysis of data from the Kids B-LONG, B-LONG, and BYOND studies. A range of PROs were used to describe in depth the evolution of QoL, pain- and activity-related domains and questions for pediatric, adolescent, and adult patients with hemophilia B treated with rFIXFc prophylaxis.

## Methods

### Study design and patient population

The study designs of Kids B-LONG, B-LONG, and B-YOND have been previously published.^17–19^ Briefly, in Kids B-LONG, previously treated (with any recombinant or plasma-derived FIX product) children [<12 years of age with ⩾50 prior exposure days (EDs) to FIX] with severe hemophilia B (defined as endogenous FIX ⩽2 IU/dl) were given prophylactic rFIXFc once weekly with a starting dose of 50–60 IU/kg, up to a maximum dose of 100 IU/kg and a maximum frequency of twice weekly.^17^ In B-LONG, previously treated adult and adolescent patients (⩾12 years of age with ⩾100 prior EDs to FIX) with severe hemophilia B were assigned to one of four treatment arms: weekly dose-adjusted rFIXFc prophylaxis (50 IU/kg starting dose), interval-adjusted rFIXFc prophylaxis (100 IU/kg every 10 days to start), on-demand treatment (20–100 IU/kg for bleeding episodes, with the dose-adjusted depending on bleeding severity), or treatment as part of perioperative care.^18^

**Figure 1.**
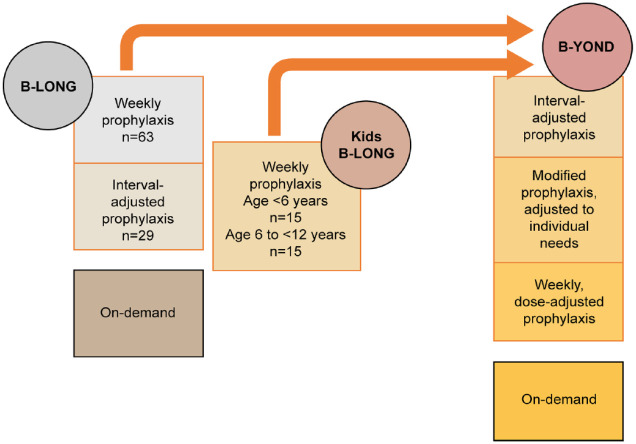
Study design. Patients could receive rFIXFc for perioperative treatment in all three studies: on-demand treatment was only for patients 12 years and above in B-YOND. rFIXFc, recombinant factor IX Fc fusion protein.

The B-YOND study enrolled eligible subjects who completed either B-LONG or Kids B-LONG (Figure 1).^19^ Patients received weekly prophylaxis (20–100 IU/kg every 7 days), individualized interval prophylaxis (100 IU/kg every 8–16 days or twice monthly), modified prophylaxis (dosing further modified to meet the needs of individual subjects), or on-demand (on-demand; only subjects ⩾12 years of age) treatment. Switching treatment regimen was permitted upon enrollment or any time during B-YOND at the investigator’s discretion to achieve the lowest effective dose resulting in an FIX trough level of 1–3 IU/dl along with dose adjustments to target trough levels up to 5 IU/dl (or >5 IU/dl for modified prophylaxis) if warranted by bleed history or activity level.

### PRO measures

Pain, physical functioning and activity were assessed on an approximately 6-month basis by Haem-A-QoL (adults only, ⩾18 years) based on the validated performances of these questionnaires in different age groups, EQ-5D-3L (adults and adolescents, ⩾12 years), EQ-5DY (pediatric patients, <12 years), HEMO-SAT (pediatric patients only, <12 years), and CHO-KLAT (pediatric patients and their caregivers only, <12 years; Table 1). The age of patients at baseline determined the age-specific instrument to be used. Haem-A-QoL responses were grouped into either never/rarely/seldom or sometimes/often/all the time; EQ-5D-3L responses were grouped into either no problem or some/severe problems; HEMO-SAT responses were grouped into either totally agree/somewhat agree or neither agree nor disagree/somewhat disagree/totally disagree; and CHO-KLAT responses were grouped into either never/rarely or sometimes/often/always.

**Table 1.** Study endpoints.

EQ-5D (<12 years)^a^	Haem-A-QoL (⩾18 years)	HEMO-SAT (<12 years)	CHO-KLAT (<12 years)
EQ-5D rating of usual activities (discrete) EQ-5D rating of pain/discomfort	**Physical health domain:** - My swellings hurt - I had pain in my joints - It was painful for me to move - I had difficulty walking as far as I wanted **Sport and leisure domain:** - I had to avoid sports that I like because of my hemophilia - I had to avoid sports like football - I played sports just as much as others - I didn’t have the freedom to travel where I wanted - It was necessary for me to plan everything in advance **Treatment domain** - I was annoyed about the amount of time spent having the injections **All subdomain scores** **Total score**	**Ease and convenience domain:** - Treatment does not interfere with our everyday life - I am satisfied with the volume of the infusion - I am satisfied with the way that the medication is administered - I am satisfied with how often my son must be infused **Efficacy domain:** - I am satisfied with the activities he is allowed to do with his treatment **General satisfaction domain:** - I am satisfied with his medication	**The child self-report version:** - It bothered me that I couldn’t play the sports that I like - I liked playing out with my friends - I felt like I had some control of my life - The treatment I got was okay - Factor injections were a bother - I got upset with my limits in physical activity - Home injections made my life easier **The caregiver proxy report version:** - It bothered my son that he couldn’t play the sports that he likes - My son liked playing out with his friends - My son felt like he had some control of his life - The treatment my son got was okay - Factor injections were a bother for my son - My son got upset with his limits in physical activity - Home injections made my son’s life easier

CHO-KLAT, Canadian Haemophilia Outcomes–Kids’ Life Assessment Tool; EQ-5D, European Quality of Life Five Dimension; Haem-A-QoL, Hemophilia Quality of Life Questionnaire for Adults; HEMO-STAT, Hemophilia-specific Treatment Satisfaction Questionnaire.

a Data for ⩾12 years only available for BYOND.

### Statistical analysis

Patients were followed from Kids B-LONG or B-LONG, into B-YOND, and until they completed the study (end of study) or discontinued. While most patients continued through to B-YOND, not all patients did, and therefore the end of study could occur during either Kids B-LONG, B-LONG, or B-YOND. No values were recorded for EQ-5D-3L in adults and adolescents during the B-LONG study as this was not part of the protocol; therefore, the BYOND 6-month values were used as a baseline. The main analysis included patients who started weekly dose-adjusted or interval-adjusted rFIXFc prophylaxis (adults and adolescents only) and completed the Haem-A-QoL and EQ-5D-3L questionnaires in the adult and adolescent population; and HEMO-SAT, CHO-KLAT, and EQ-5DY in the pediatric population. The sensitivity analysis included all patients who started prophylaxis in Kids B-LONG or B-LONG and were followed until switch to modified prophylaxis (or end of follow-up as in the main analysis).

Descriptive analysis of categorical variables was reported using number of subjects and percentages; continuous variables were reported, mean, standard deviation, median, percentiles (25.00% and 75.00%), minimum, and maximum. Change over time was assessed by comparing individual patient values at each time point with their baseline level (paired values), as patient numbers changed during the course of the study. For categorical variables, paired McNemar tests were used (a continuity correction was applied when any of the cell counts were <). The Wilcoxon signed rank test was used for continuous variables. All data processing and analyses were to be performed using SAS software version 9.4 or higher (SAS Institute Inc., Cary, NC, USA) and RStudio Software version 4.0.4.

## Results

### Patient population

Patient demographics have been published previously.^17–19^ There was a fairly even split between adults and adolescents by previous treatment regimens prior to receiving rFIXFc, whereas all children were previously treated with prophylaxis (Table 2). Ninety-two adult and adolescent patients that started in the B-LONG study were assessed, with a median (range) duration of follow-up of 58.9 (0.0–78.4) months. Thirty pediatric patients that started in the Kids B-LONG study were assessed, with a median (min–max) duration of follow-up of 36.7 (9.0–59.9) months. In the adult and adolescent population (*n* = 92), 14 patients were excluded from the sensitivity analysis (total sensitivity analysis population, *n* = 78) due to switching to modified prophylaxis. In the pediatric population (*n* = 30), two patients were excluded from the sensitivity analysis (total sensitivity analysis population, *n* = 28) due to switching to modified prophylaxis.

**Table 2.** Patient demographics.

	Adults and adolescents (⩾12 years)	Children (<12 years)
	*N* = 92	*N* = 30
Age, years [mean (SD)]	32.77 (14.14)	5.47 (3.16)
Weight, kg [mean (SD)]	76.41 (20.57)	25.01 (11.23)
Prior FIX regimen [*n* (%)]		
On-demand	43 (47.25)	0 (0.00)
Prophylaxis	48 (52.75)	30 (100.00)
Duration of follow-up, months [median (range)]	58.9 (0.0–78.4)	36.7 (9.0–59.9)

FIX, factor IX; SD, standard deviation.

### Adults and adolescents

#### Haem-A-QoL total and sub domain scores

**Figure 2.**
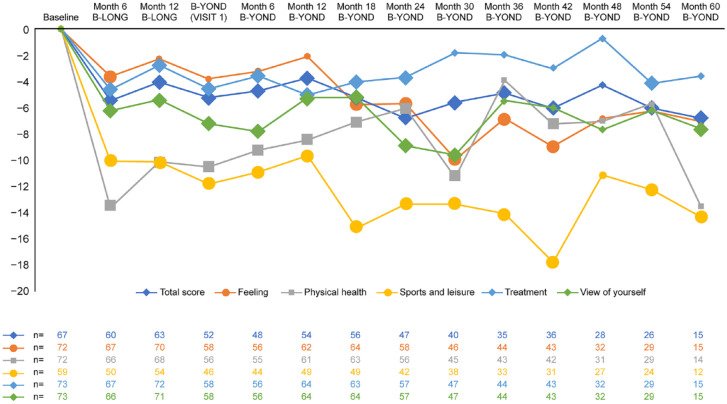
Change from baseline in Haem-A-QoL total score and subdomains score in patients originally enrolled in B-LONG. Large data markers indicate significant results (*p* < 0.05 comparison with B-LONG baseline); subdomain scores with no significant changes are not included. Haem-A-QoL, Haemophilia Quality of Life Questionnaire for Adults.

**Figure 3.**
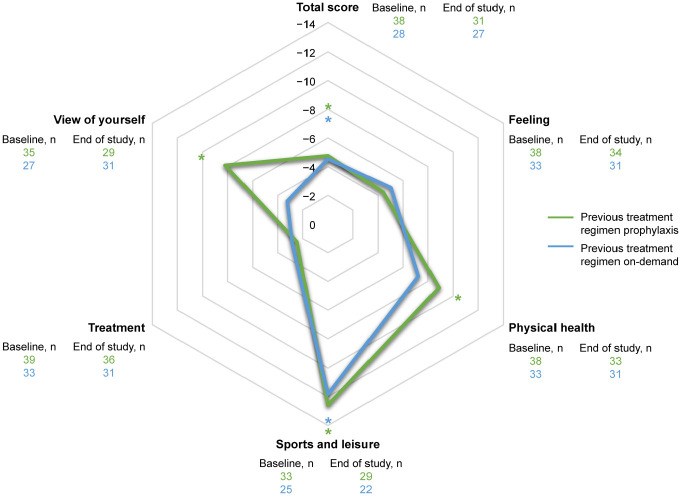
Change from baseline to end of study in Haem-A-QoL total and subdomains scores in patients originally enrolled in B-LONG. Haem-A-QoL, Haemophilia Quality of Life Questionnaire for Adults. *Significant results (*p* < 0.05 comparison with B-LONG baseline).

Statistically significant and sustained improvements were observed in mean [standard deviation (SD)] Haem-A-QoL total score, with a reduction of 4.45 (10.53; *n* = 59; *p* ⩽ 0.01) at the end of study compared with the baseline. The mean (SD) reduction from baseline to end of study (which could refer to any time point during B-LONG or B-YOND) in the Haem-A-QoL subdomain ‘feeling’ was 4.26 (20.69; *n* = 66; *p* = 0.058); in the subdomain ‘physical health’ was 9.1 (22.58; *n* = 65; *p* = 0.001); in the subdomain ‘sports and leisure’ score was 11.25 (25.5; *n* = 52; *p* ⩽ 0.01); in the subdomain ‘treatment’ was 2.69 (12.84; *n* = 68; *p* = 0.05); and in the subdomain ‘view of self’ was 5.81 (17.42; *n* = 68; *p* = 0.002) (Supplemental Table 1; Figures 2 and 3). These results were confirmed by the sensitivity analysis (Supplemental Table 2).

No significant difference at any time point was shown for family planning, partnership, and sexuality and future (Supplemental Table 1).

#### Haem-A-QoL items

**Figure 4.**
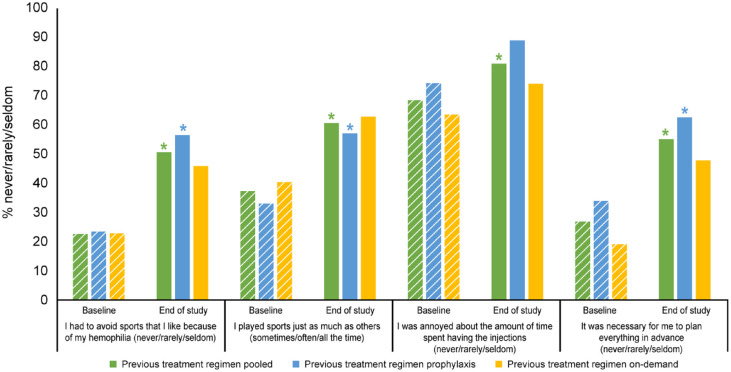
Satisfaction levels in Haem-A-QoL items (patients originally enrolled in B-LONG) reporting ‘never/rarely/seldom’. *Significant results (*p* < 0.05 comparison with B-LONG baseline). Haem-A-QoL, Haemophilia Quality of Life Questionnaire for Adults.

**Figure 5.**
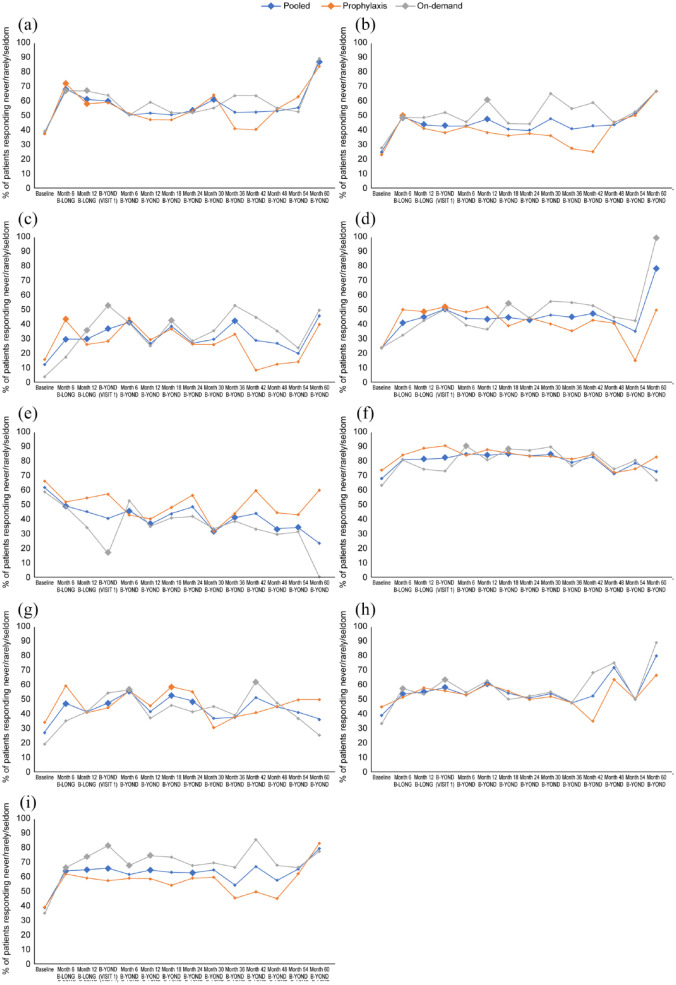
Change in satisfaction levels in Haem-A-QoL items (patients originally enrolled in B-LONG) reporting ‘never/rarely/seldom’. (a) I had difficulty walking as far as I wanted to, (b) I had pain in my joints, (c) I had to avoid sports like football, (d) I had to avoid sports that I like because of my hemophilia, (d) I played sports just as much as others, (f) I was annoyed about the amount of time spent having the injections, (g) It was necessary for me to plan everything in advance, (h) It was painful for me to move, (i) My swellings hurt. Large data markers indicate significant results (*p* < 0.05 comparison with B-LONG baseline); items with no significant changes are not included. Haem-A-QoL, Haemophilia Quality of Life Questionnaire for Adults.

Significant and sustained improvements were found in Haem-A-QoL items, ‘I had to avoid sports that I like because of my hemophilia’, ‘I played sports just as much as others’, ‘I was annoyed about the amount of time spent having the injections’ and ‘It was necessary for me to plan everything in advance’ (Figure 4; Supplemental Table 3). Significant improvements were observed in Haem-A-QoL items ‘I had to avoid sports like football’, ‘I had difficulty walking as far as I wanted to’, ‘It was painful for me to move’, ‘My swellings hurt’, and ‘I had pain in my joints’, but baseline to end of study were not significant (Figure 5; Supplemental Table 3). No significant improvements were observed in the Haem-A-QoL item ‘I didn’t have the freedom to travel where I wanted’ (Supplemental Table 3).

### Pediatric patients

#### HEMO-SAT results

**Figure 6.**
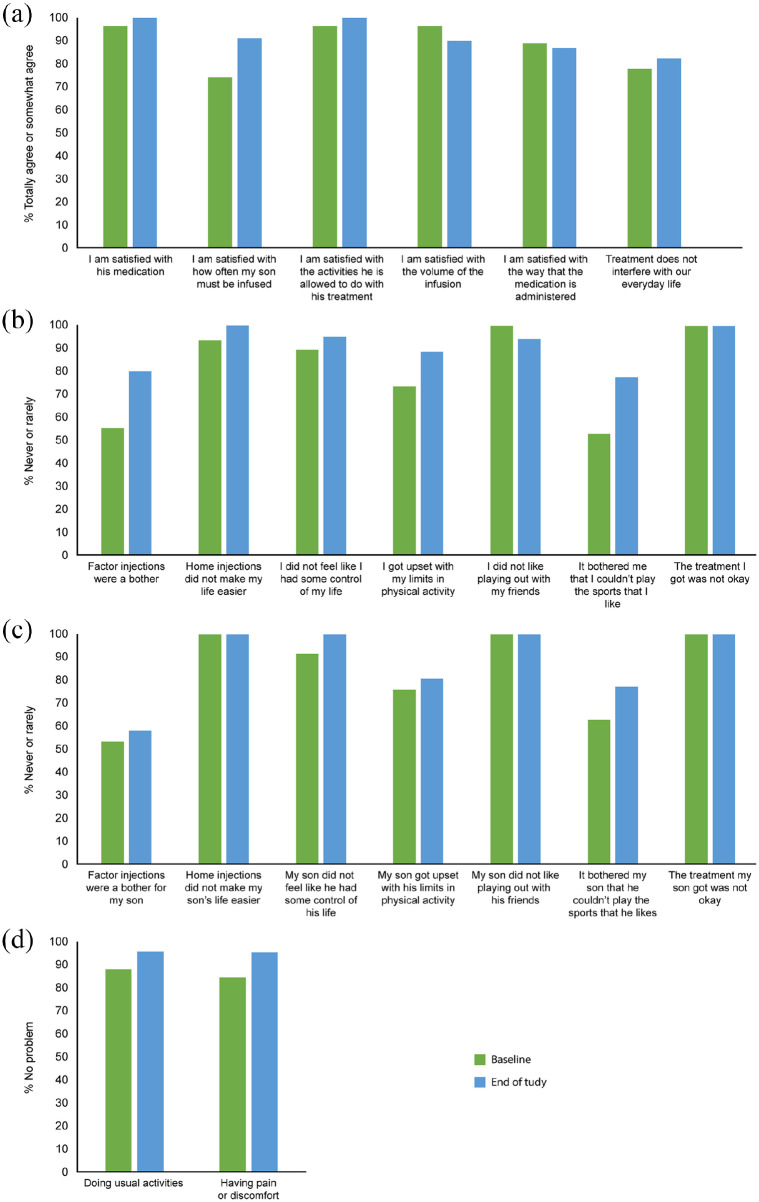
Levels of satisfaction in pediatric patients (originally enrolled in Kids B-LONG) at baseline and end of study for (a) HEMO-SAT caregiver’s perspective, (b) CHO-KLAT child’s perspective, (c) CHO-KLAT caregiver’s perspective, and (d) EQ-5D child’s perspective. CHO-KLAT, Canadian Haemophilia Outcomes–Kids’ Life Assessment Tool; EQ-5D, European Quality of Life Five Dimension; HEMO-SAT, Hemophilia-specific Treatment Satisfaction Questionnaire.

A high proportion of caregivers of children reported satisfaction in all items at baseline, which was sustained throughout the study (Figure 6). A significant change from baseline was demonstrated at the 12-month B-YOND visit in the item ‘I am satisfied with how often my son must be infused’. No significant results were found in any other item.

#### CHO-KLAT results

A significant change from baseline was found at the 12-month B-YOND visit in the item ‘Factor injections were a bother’, from the child’s perspective (data not shown), although there were no significant changes for ‘Factor injections were a bother for my son’ from the caregiver’s perspective. No significant results were observed in any other item, although for all items apart from ‘It bothered me that I couldn’t play the sports that I like’ and ‘It bothered my son that he couldn’t play the sports that he likes’, initial satisfaction levels at baseline were high (Figure 6).

#### EQ-5DY pain/discomfort and usual activities

The percentage of pediatric patients directly reporting having no problem with pain/discomfort or usual activities was maintained over the course of the study, but no significant improvements were demonstrated. The proportion of patients with no problems at baseline was high (Figure 6), with only 12.00% of patients reporting ‘some or severe problems’ with *usual activities* and 16.00% of patients reporting ‘some or severe problems’ with *pain/discomfort*.

## Discussion

In this *post hoc* analysis of Kids B-LONG, B-LONG, and B-YOND data, PROs assessing QoL measures of pain and physical activity demonstrated at least a maintenance of baseline scores and, in some incidences, a significant and sustained improvement over approximately 5 years. In the pivotal studies upon which this *post hoc* analysis is based, bleeding rates were reduced from the levels recorded prior to the parent study,^17,18^ and remained low in the study extension.^19^ Given the well-documented link between bleeding and pain, the results of this study were expected and add to the already published data that demonstrate the ability of rFIXFc to improve HRQoL, with reduced dosing frequency and a good tolerance profile.^17–23^

Haem-A-QoL improvements in adults and adolescents were maintained over time for the total score and the domains sports and leisure, physical health, feeling, and view of self; confirming both the results from the initial B-LONG study,^21^ and the interim analysis of the BYOND study.^22^ There is a paucity of data on the impact of prophylaxis with EHL FIX products on HRQoL in patients with hemophilia B,^24^ making it difficult to ratify these results against previous studies. As the emphasis shifts away from focusing solely on bleed prevention toward a more holistic approach for treating hemophilia B,^14^ the impact of successful treatment on HRQoL will be revealed.

There was consistent improvement and maintained benefit of prophylaxis with rFIXFc in adults and adolescents on the Haem-A-QoL items relating to pain and activities (sports/walking). An increase over time was observed in the proportion of adult and adolescent patients never/rarely/seldom reporting difficulties walking as far as they wanted, pain in their joints, pain when moving, painful swellings, and avoidance of sport.

The lack of adult and adolescent EQ-5D-3L results from B-LONG meant that any initial improvements from rFIXFc prophylaxis in the first 18 months were not captured, preventing any analysis of change from baseline. However, the EQ-5D-3L results for adults and adolescents showed that the proportion of patients reporting pain/discomfort and problems with usual activities was sustained over time from B-YOND month 6 to B-YOND end of study (data not shown). Similarly, the EQ-5D-3L results for pediatric patients showed minimal change from baseline to end of study, reflecting the maintenance of the relatively high baseline scores. The low number of pediatric patients reporting problems at baseline resulted in a ceiling effect, limiting that variability of the results, and leaving little room for improvement.

The results of this *post hoc* analysis suggest that there is a general improvement in levels of pain and physical activity upon initiating prophylactic treatment with rFIXFc, which is maintained, with long-term improvements observed in patient esteem and satisfaction. Therefore, successful, long-term, prophylactic treatment of patients with hemophilia B may well be pivotal in the goal of ‘living with a hemophilia-free mind’.^14^

In pediatric patients treated with rFIXFc prophylaxis, high satisfaction levels were maintained, with improvements seen relating to factor injections from both the caregiver (HEMO-SAT) and child’s (CHO-KLAT) viewpoint. The lack of significant improvement may be a result of the ceiling effect, or it could be attributed to age, as pain has a tendency to increase and QoL has a tendency to decrease in the adult population compared with the pediatric population.^25,26^ This is unsurprising given that the progression of joint damage, and therefore the subsequent joint pain, can be delayed but not halted.^27^ Further research is needed to understand the association between declining HRQoL and age of patients.

This study had limitations, including the lack of a comparator and the decline in patient numbers over time, primarily throughout B-YOND due to the planned end of study upon rFIXFc becoming commercially available, which was at different timepoints in the participating countries. In addition, the high baseline scores and small sample size for pediatric patients limited the conclusions that could be drawn. There are also limitations related to the use of PROs and the validity of grouping reporting levels. Assessing pain and physical activity with PROs, where responses are subjective and are not set against an objective baseline, limits the ability to draw absolute conclusions. In addition, the averaging of whole integers results in numerical scores that cannot distinguish between a patient who always has a problem on one item and a patient who occasionally has problems across many items, limiting the potential to analyze individual domains from a patient’s overall scores.^28^

## Conclusion

Long-term rFIXFc prophylaxis reduces perceived pain and increases levels of physical activity in adult and adolescent patients with hemophilia B and maintains high QoL scores in pediatric patients. The analyses of subdomains within PRO instruments highlight the relevance of pain and physical health (mobility, ability to perform daily life activities and sports) as the concepts most sensitive to change with prophylaxis treatment and indicate the potential for improving long-term QoL in patients with hemophilia B.

## Supplemental Material

sj-docx-1-tah-10.1177_20406207231170701 – Supplemental material for Recombinant factor IX Fc prophylaxis reduces pain and increases levels of physical activity, with sustained, long-term improvements in patients with hemophilia B: post hoc analysis of phase III trials using patient-reported outcomes Click here for additional data file. Supplemental material, sj-docx-1-tah-10.1177_20406207231170701 for Recombinant factor IX Fc prophylaxis reduces pain and increases levels of physical activity, with sustained, long-term improvements in patients with hemophilia B: post hoc analysis of phase III trials using patient-reported outcomes by Jan Astermark, Cédric Hermans, Monia Ezzalfani, Alaeddine Sidhom, Sylvaine Barbier, Nana Kragh, Aletta Falk and Daniel Eriksson in Therapeutic Advances in Hematology

## Declarations

**Ethics approval and consent to participate:** This analysis was based on data from previously published clinical trials. These had been performed in accordance with the Declaration of Helsinki and local regulations. Protocols were approved by the authorities and the ethics committees of the institutions involved. Signed informed consent was obtained from patients or patients’ guardians prior to participation.

**Consent for publication:** As this study used anonymized data from the previously published trials, further informed consent was not required.

**Availability of data and materials:** Sobi is committed to responsible and ethical sharing of data on participant level and summary data for medicines and indications approved by EMA and/or FDA while protecting individual participant integrity and compliance with applicable legislation. Data access will be granted in response to qualified research requests. All requests are evaluated by a cross-functional panel of experts within Sobi, and a decision on sharing will be based on the scientific merit and feasibility of the research proposal, maintenance of personal integrity, and commitment to publication of the results. To request access to study data, a data sharing request form (available on www.sobi.com) should be sent to medical.info@sobi.com. Further information on Sobi’s data sharing policy and process for requesting access can be found at: https://www.sobi.com/en/policies.
