# Supplementary material for: Recombinant factor IX Fc prophylaxis reduces pain and increases levels of physical activity, with sustained, long-term improvements in patients with hemophilia B: post hoc analysis of phase III trials using patient-reported outcomes
Source: Ther Adv Hematol. 2023 May 29;14:20406207231170701. doi: 10.1177/20406207231170701 (PMC10240555; doi:10.1177/20406207231170701)
Supplement: sj-docx-1-tah-10.1177_20406207231170701 – Supplemental material for Recombinant factor IX Fc prophylaxis reduces pain and increases levels of physical activity, with sustained, long-term improvements in patients with hemophilia B: post hoc analysis of phase III trials using patient-reported outcomes [file sj-docx-1-tah-10.1177_20406207231170701.docx]

**Supplement**

**TABLE 1.** Haem-A-QoL total scores and subdomain scores (patients originally enrolled in B-LONG).

| Subdomain* | Patient population^†^ |  | Baseline/  B-LONG | Month 6  B-LONG | Month 12  B-LONG | B-YOND  (VISIT 1) | Month 12/  B-YOND | Month 24/  B-YOND | Month 36/  B-YOND | Month 48/  B-YOND | End of study^‡^ |
| --- | --- | --- | --- | --- | --- | --- | --- | --- | --- | --- | --- |
| Total score | Previous treatment regimen prophylaxis | n | 38 | 32 | 36 | 32 | 30 | 26 | 17 | 11 | 31 |
|  |  | Mean (SD) | 32.60 (16.51) | -5.16 (7.4) | -2.54 (10.43) | -3.60 (9.55) | -2.45 (11.22) | -5.25 (8.07) | -2.84 (10.72) | -0.8 (13.87) | -4.77 (9.81) |
|  |  | P-value |  | 0.001 | 0.091 | 0.013 | 0.213 | 0.004 | 0.616 | 0.944 | 0.001 |
|  | Previous treatment regimen on-demand | n | 28 | 27 | 26 | 19 | 23 | 21 | 18 | 16 | 27 |
|  |  | Mean (SD) | 32.90 (14.16) | -6.16 (9.5) | -6.98 (8.48) | -9.19 (8.53) | -5.68 (10.51) | -8.66 (8.12) | -6.8 (12.81) | -7.49 (9.30) | -4.55 (11.4) |
|  |  | P-value |  | 0.004 | 0.001 | 0.001 | 0.021 | 0 | 0.059 | 0.014 | 0.038 |
|  | Total | n | 67 | 60 | 63 | 52 | 54 | 47 | 35 | 28 | 59 |
|  |  | Mean (SD) | 32.90 (15.39) | -5.45 (8.39) | -4.05 (10.15) | -5.24 (9.95) | -3.75 (10.85) | -6.78 (8.18) | -4.88 (11.84) | -4.26 (11.72) | -4.45 (10.53) |
|  |  | P-value |  | ≤ 0.01 | 0.001 | ≤ 0.01 | 0.013 | ≤ 0.01 | 0.05 | 0.076 | ≤ 0.01 |
| Dealing with hemophilia | Previous treatment regimen prophylaxis | n | 39 | 33 | 39 | 34 | 33 | 31 | 21 | 11 | 35 |
|  |  | Mean (SD) | 25.64 (25.96) | -3.79 (29.62) | -4.27 (26.06) | -4.41 (26.85) | -4.55 (18.17) | -2.69 (28.50) | 4.76 (32.76) | -4.55 (22.47) | -4.05 (21.14) |
|  |  | P-value |  | 0.379 | 0.205 | 0.241 | 0.205 | 0.284 | 0.552 | 1 | 0.38 |
|  | Previous treatment regimen on-demand | n | 33 | 33 | 32 | 23 | 27 | 24 | 22 | 19 | 31 |
|  |  | Mean (SD) | 24.24 (16.58) | -5.56 (24.62) | 0.26 (26.81) | 1.45 (30.11) | -11.42 (22.78) | -8.68 (22.05) | -11.36 (22.05) | -10.09 (21.62) | -9.41 (22.33) |
|  |  | P-value |  | 0.121 | 0.775 | 0.97 | 0.028 | 0.078 | 0.006 | 0.045 | 0.074 |
|  | Total | n | 73 | 67 | 72 | 58 | 61 | 56 | 44 | 31 | 67 |
|  |  | Mean (SD) | 25.11 (21.89) | -4.60 (26.84) | -2.66 (26.38) | -2.59 (28.15) | -7.51 (20.34) | -5.21 (25.60) | -3.03 (28.48) | -8.87 (21.83) | -6.47 (21.56) |
|  |  | P-value |  | 0.083 | 0.189 | 0.252 | 0.009 | 0.042 | 0.09 | 0.07 | 0.053 |
| Family planning | Previous treatment regimen prophylaxis | n | 31 | 20 | 24 | 19 | 20 | 17 | 8 | 8 | 19 |
|  |  | Mean (SD) | 17.74 (25.96) | -2.40 (12.15) | -0.43 (13.56) | 2.3 (13.06) | 2.29 (11.90) | -4.9 (15.83) | -4.43 (14.71) | 2.08 (16.40) | -1.64 (10.45) |
|  |  | P-value |  | 0.433 | 0.875 | 0.514 | 0.797 | 0.292 | 0.584 | 1 | 0.435 |
|  | Previous treatment regimen on-demand | n | 19 | 16 | 18 | 11 | 11 | 14 | 11 | 9 | 14 |
|  |  | Mean (SD) | 21.49 (24.16) | -4.43 (19.78) | 2.08 (11.43) | 1.52 (14.13) | 3.98 (16.36) | -8.48 (26.93) | -5.49 (28.69) | -8.33 (20.73) | -2.53 (16.18) |
|  |  | P-value |  | 0.363 | 0.72 | 0.916 | 1 | 0.289 | 1 | 0.181 | 1 |
|  | Total | n | 51 | 37 | 43 | 31 | 32 | 31 | 20 | 18 | 34 |
|  |  | Mean (SD) | 18.79 (25.00) | -1.86 (17.85) | 1.21 (13.00) | 2.76 (13.64) | 2.80 (13.19) | -6.52 (21.24) | -2.92 (24.57) | -3.24 (18.46) | -1.59 (13.01) |
|  |  | P-value |  | 0.498 | 0.841 | 0.451 | 0.664 | 0.105 | 0.944 | 0.248 | 0.497 |
| Feeling | Previous treatment regimen prophylaxis | n | 38 | 33 | 38 | 34 | 33 | 32 | 22 | 11 | 34 |
|  |  | Mean (SD) | 22.37 (22.07) | -5.49 (17.38) | -2.3 (19.95) | -3.49 (20.54) | -1.33 (19.87) | -3.52 (17.89) | -1.7 (25.82) | 2.27 (25.81) | -4.41 (17.03) |
|  |  | P-value |  | 0.035 | 0.282 | 0.13 | 0.585 | 0.125 | 0.269 | 1 | 0.07 |
|  | Previous treatment regimen on-demand | n | 33 | 33 | 31 | 23 | 28 | 25 | 22 | 20 | 31 |
|  |  | Mean (SD) | 25.76 (17.03) | -2.46 (21.59) | -3.23 (16.21) | -5.71 (17.36) | -4.46 (20.33) | -9.25 (23.25) | -11.93 (23.93) | -14.06 (19.44) | -5.04 (24.07) |
|  |  | P-value |  | 0.308 | 0.304 | 0.099 | 0.253 | 0.138 | 0.035 | 0.023 | 0.22 |
|  | Total | n | 72 | 67 | 70 | 58 | 62 | 58 | 44 | 32 | 66 |
|  |  | Mean (SD) | 24.31 (19.92) | -3.64 (19.56) | -2.32 (18.40) | -3.88 (19.41) | -2.12 (20.46) | -5.71 (20.39) | -6.82 (25.14) | -6.84 (23.94) | -4.26 (20.69) |
|  |  | P-value |  | 0.045 | 0.198 | 0.053 | 0.352 | 0.038 | 0.018 | 0.164 | 0.058 |
| Future | Previous treatment regimen prophylaxis | n | 38 | 32 | 38 | 33 | 32 | 31 | 21 | 11 | 34 |
|  |  | Mean (SD) | 37.89 (21.98) | -3.44 (9.20) | -3.55 (17.47) | -3.64 (15.87) | -0.47 (14.39) | -4.35 (16.97) | -3.1 (20.22) | -1.82 (17.93) | -5.88 (18.71) |
|  |  | P-value |  | 0.07 | 0.248 | 0.213 | 0.598 | 0.044 | 0.323 | 1 | 0.021 |
|  | Previous treatment regimen on-demand | n | 32 | 33 | 32 | 23 | 28 | 25 | 22 | 19 | 31 |
|  |  | Mean (SD) | 40.47 (21.26) | 0.00 (14.47) | -4.06 (14.11) | -6.09 (15.37) | -2.32 (16.07) | -2.80 (14.73) | -1.36 (19.22) | -2.63 (18.06) | 1.29 (18.06) |
|  |  | P-value |  | 1 | 0.179 | 0.07 | 0.549 | 0.65 | 0.648 | 0.916 | 1 |
|  | Total | n | 71 | 66 | 71 | 57 | 61 | 56 | 44 | 31 | 66 |
|  |  | Mean (SD) | 38.94 (21.41) | -1.97 (12.31) | -3.17 (16.63) | -3.86 (16.53) | -1.31 (14.97) | -3.66 (15.88) | -2.27 (19.27) | -2.26 (17.41) | -1.97 (18.9) |
|  |  | P-value |  | 0.196 | 0.136 | 0.079 | 0.447 | 0.056 | 0.338 | 0.851 | 0.128 |
| Partnership and sexuality | Previous treatment regimen prophylaxis | n | 37 | 33 | 37 | 33 | 33 | 31 | 21 | 11 | 34 |
|  |  | Mean (SD) | 16.22 (27.77) | 0.76 (20.02) | -0.90 (17.32) | 0 (15.45) | -1.77 (24.27) | -2.96 (20.47) | -9.52 (19.59) | -8.33 (24.15) | -1.47 (19.94) |
|  |  | P-value |  | 1 | 0.776 | 1 | 0.969 | 0.953 | 0.063 | 0.371 | 0.599 |
|  | Previous treatment regimen on-demand | n | 32 | 32 | 31 | 22 | 26 | 23 | 20 | 19 | 29 |
|  |  | Mean (SD) | 11.98 (23.56) | 3.91 (16.39) | 0.54 (12.53) | -3.03 (11.37) | 5.45 (16.49) | 5.07 (19.26) | 3.33 (24.39) | -0.44 (10.20) | 1.15 (15.22) |
|  |  | P-value |  | 0.26 | 0.721 | 0.268 | 0.407 | 0.609 | 0.833 | 0.892 | 0.838 |
|  | Total | n | 70 | 66 | 69 | 56 | 60 | 55 | 42 | 31 | 64 |
|  |  | Mean (SD) | 14.76 (25.97) | 1.52 (19.22) | -0.48 (15.25) | -1.49 (13.95) | 0.56 (22.17) | -0.45 (21.12) | -4.37 (23.59) | -3.76 (16.64) | 0.26 (18.18) |
|  |  | P-value |  | 0.646 | 0.895 | 0.398 | 0.858 | 0.627 | 0.116 | 0.261 | 0.831 |
| Physical health | Previous treatment regimen prophylaxis | n | 38 | 32 | 37 | 33 | 33 | 31 | 21 | 10 | 33 |
|  |  | Mean (SD) | 44.93 (26.31) | -13.67 (17.17) | -8.75 (20.16) | -7.99 (20.32) | -5.45 (21.99) | -5.24 (17.78) | 0.42 (23.76) | 1.50 (28.48) | -8.86 (21.5) |
|  |  | P-value |  | ≤ 0.01 | 0.006 | 0.015 | 0.123 | 0.132 | 0.887 | 0.866 | ≤ 0.01 |
|  | Previous treatment regimen on-demand | n | 33 | 33 | 30 | 22 | 27 | 25 | 22 | 20 | 31 |
|  |  | Mean (SD) | 43.94 (21.38) | -13.03 (17.64) | -12.75 (20.03) | -15.57 (21.13) | -11.2 (27.42) | -7.1 (19.12) | -8.12 (21.37) | -9.44 (23.4) | -7.22 (21.13) |
|  |  | P-value |  | ≤ 0.01 | 0.003 | 0.005 | 0.085 | 0.038 | 0.052 | 0.026 | 0.065 |
|  | Total | n | 72 | 66 | 68 | 56 | 61 | 56 | 43 | 31 | 65 |
|  |  | Mean (SD) | 45.03 (24.28) | -13.67 (17.35) | -10.17 (20.14) | -10.56 (20.89) | -8.48 (24.57) | -6.07 (18.24) | -3.95 (22.71) | -7.06 (25.81) | -9.1 (22.58) |
|  |  | P-value |  | ≤ 0.01 | ≤ 0.01 | ≤ 0.01 | 0.012 | 0.009 | 0.197 | 0.063 | 0.001 |
| Sports and leisure | Previous treatment regimen prophylaxis | n | 33 | 26 | 31 | 29 | 26 | 23 | 15 | 10 | 29 |
|  |  | Mean (SD) | 56.55 (25.49) | -10.82 (24.42) | -5.12 (23.94) | -6.64 (23.53) | -11.11 (27.75) | -11.03 (19.47) | -12.33 (30.05) | -5.75 (18.59) | -12.59 (24.61) |
|  |  | P-value |  | 0.055 | 0.274 | 0.161 | 0.056 | 0.014 | 0.149 | 0.67 | 0.004 |
|  | Previous treatment regimen on-demand | n | 25 | 23 | 22 | 16 | 22 | 18 | 17 | 16 | 22 |
|  |  | Mean (SD) | 60.05 (20.49) | -11.03 (17.99) | -20 (18.16) | -25.08 (17.34) | -10.28 (22.8) | -19.44 (17.96) | -19.34 (28.07) | -17.50 (25.82) | -11.82 (25.38) |
|  |  | P-value |  | 0.011 | ≤ 0.01 | 0.001 | 0.041 | 0.003 | 0.108 | 0.041 | 0.013 |
|  | Total | n | 59 | 50 | 54 | 46 | 49 | 42 | 33 | 27 | 52 |
|  |  | Mean (SD) | 57.75 (23.25) | -10.1 (21.97) | -10.16 (24.05) | -11.82 (24.69) | -9.69 (26.10) | -13.42 (20.64) | -14.13 (30.39) | -11.20 (24.94) | -11.25 (25.50) |
|  |  | P-value |  | 0.003 | 0.003 | 0.003 | 0.012 | 0.001 | 0.037 | 0.116 | ≤ 0.01 |
| Treatment | Previous treatment regimen prophylaxis | n | 39 | 33 | 39 | 34 | 35 | 31 | 22 | 11 | 36 |
|  |  | Mean (SD) | 29.17 (13.63) | -4.79 (11.7) | -2.29 (13.53) | -4.02 (10.92) | -3.16 (13.32) | -3.77 (12.62) | -0.2 (14.44) | 3.57 (13.73) | -2.47 (10.64) |
|  |  | P-value |  | 0.026 | 0.292 | 0.036 | 0.075 | 0.036 | 0.43 | 0.621 | 0.12 |
|  | Previous treatment regimen on-demand | n | 33 | 33 | 32 | 23 | 28 | 25 | 22 | 20 | 31 |
|  |  | Mean (SD) | 32.39 (17.06) | -4.71 (19.44) | -3.81 (16.16) | -6.11 (16.03) | -7.17 (11.12) | -4 (16.05) | -3.69 (20.74) | -3.75 (15.66) | -2.82 (15.36) |
|  |  | P-value |  | 0.247 | 0.17 | 0.077 | 0.006 | 0.213 | 0.348 | 0.232 | 0.305 |
|  | Total | n | 73 | 67 | 72 | 58 | 64 | 57 | 44 | 32 | 68 |
|  |  | Mean (SD) | 31.08 (15.62) | -4.58 (15.85) | -2.76 (14.69) | -4.56 (13.21) | -5.06 (12.39) | -3.7 (14.05) | -1.95 (17.75) | -0.73 (15.14) | -2.69 (12.84) |
|  |  | P-value |  | 0.026 | 0.099 | ≤ 0.01 | 0.001 | 0.018 | 0.182 | 0.292 | 0.05 |
| View of yourself | Previous treatment regimen prophylaxis | n | 39 | 33 | 38 | 34 | 35 | 31 | 22 | 11 | 36 |
|  |  | Mean (SD) | 40 (24.74) | -5.68 (13.63) | -4.74 (17.40) | -6.62 (16.22) | -5.25 (18.43) | -9.6 (16.66) | -8.18 (17.56) | -10.45 (27.52) | -8.19 (16.95) |
|  |  | P-value |  | ≤ 0.01 | 0.098 | ≤ 0.018 | 0.153 | 0.005 | 0.018 | 0.551 | ≤ 0.01 |
|  | Previous treatment regimen on-demand | n | 33 | 32 | 32 | 23 | 28 | 25 | 22 | 20 | 31 |
|  |  | Mean (SD) | 42.27 (20.47) | -7.03 (16.89) | -6.41 (18.33) | -8.48 (19.91) | -5.89 (20.77) | -8.40 (21.4) | -2.73 (19.62) | -7.31 (20.50) | -3.23 (18.10) |
|  |  | P-value |  | 0.036 | 0.032 | 0.022 | 0.275 | 0.159 | 0.732 | 0.361 | 0.394 |
|  | Total | n | 73 | 66 | 71 | 58 | 64 | 57 | 44 | 32 | 68 |
|  |  | Mean (SD) | 41.16 (22.62) | -6.25 (15.12) | -5.42 (17.6) | -7.24 (17.53) | -5.29 (19.29) | -8.9 (18.62) | -5.45 (18.61) | -7.7 (22.83) | -5.81 (17.42) |
|  |  | P-value |  | 0.001 | 0.008 | 0.001 | 0.07 | 0.002 | 0.071 | 0.336 | 0.002 |
| Work and school | Previous treatment regimen prophylaxis | n | 35 | 27 | 32 | 27 | 26 | 25 | 17 | 10 | 29 |
|  |  | Mean (SD) | 22.5 (22.65) | 1 (17.54) | 1.76 (18.60) | 1.39 (18.70) | 2.64 (18.89) | -1.75 (15.78) | 1.84 (14.78) | 2.5 (22.28) | 0.86 (15.19) |
|  |  | P-value |  | 1 | 0.759 | 1 | 0.443 | 0.565 | 0.893 | 0.854 | 0.473 |
|  | Previous treatment regimen on-demand | n | 27 | 26 | 25 | 18 | 22 | 18 | 17 | 15 | 26 |
|  |  | Mean (SD) | 25.62 (24.17) | -6.25 (14.23) | -5.67 (18.83) | -3.36 (18.33) | -6.72 (17.44) | -10.88 (15.48) | -7.97 (16.96) | -7.36 (18.50) | -2.80 (14.48) |
|  |  | P-value |  | 0.052 | 0.12 | 0.406 | 0.137 | 0.018 | 0.073 | 0.106 | 0.719 |
|  | Total | n | 63 | 54 | 58 | 46 | 49 | 44 | 35 | 26 | 56 |
|  |  | Mean (SD) | 23.68 (23.04) | -1.81 (17.01) | -0.83 (19.42) | 0.32 (19.12) | -1.11 (18.85) | -4.02 (18.96) | -2.26 (16.88) | 0.08 (26.68) | 0.04 (16.2) |
|  |  | P-value |  | 0.305 | 0.542 | 0.79 | 0.814 | 0.127 | 0.494 | 0.342 | 0.639 |

*Sum of patients for whom results are shown by previous treatment regimen may differ by 1 from the total for the two subgroups due to missing data for one patient; ^†^Treatment regimen prior to enrolling in B-LONG; ^‡^End of study could refer to any timepoint during B-LONG or B‑YOND, depending on when the patient completed the study

P-value <0.05 indicates a statistically significant difference.

Haem-A-QoL, Haemophilia Quality of Life Questionnaire for Adults; SD, standard deviation.

**TABLE 2.** Haem-A-QoL total sensitivity analysis total score and subdomain scores (patients originally enrolled in B-LONG).

|  |  | Baseline/  B-LONG | Month 6  B-LONG | Month 12  B-LONG | B-YOND  (VISIT 1) | Month 12/  B-YOND | Month 24/  B-YOND | Month 36/  B-YOND | Month 48/  B-YOND | End of study* |
| --- | --- | --- | --- | --- | --- | --- | --- | --- | --- | --- |
| Total score | n | 67 | 60 | 63 | 43 | 43 | 35 | 29 | 22 | 46 |
|  | Mean (SD)* | 32.9 (15.39) | -5.45 (8.39) | -4.05 (10.15) | -5.32 (8.91) | -2.92 (11.19) | -6.08 (7.72) | -3.24 (11.29) | -3.26 (10.80) | -3.64 (9.30) |
|  | P-value |  | ≤ 0.01 | 0.001 | ≤ 0.01 | 0.087 | ≤ 0.01 | 0.266 | 0.187 | 0.003 |
| Dealing with hemophilia | n | 73 | 67 | 72 | 49 | 50 | 44 | 37 | 25 | 53 |
|  | Mean (SD)* | 25.11 (21.89) | -4.6 (26.84) | -2.66 (26.38) | -2.55 (28.22) | -8.83 (17.12) | -4.55 (25.7) | -2.7 (30.18) | -11.67 (22.05) | -7.86 (20.89) |
|  | P-value |  | 0.083 | 0.189 | 0.213 | 0.002 | 0.057 | 0.15 | 0.025 | 0.014 |
| Family planning | n | 51 | 37 | 43 | 23 | 25 | 22 | 16 | 14 | 26 |
|  | Mean (SD)* | 18.79 (25.00) | -1.86 (17.85) | 1.21 (13.00) | 4.89 (13.60) | 3.33 (13.53) | -3.88 (21.68) | 0.65 (24.2) | -0.15 (12.02) | -0.72 (10.75) |
|  | P-value |  | 0.498 | 0.841 | 0.193 | 0.68 | 0.672 | 0.178 | 0.423 | 0.666 |
| Feeling | n | 72 | 67 | 70 | 49 | 50 | 46 | 37 | 25 | 52 |
|  | Mean (SD)* | 24.31 (19.92) | -3.64 (19.56) | -2.32 (18.40) | -5.23 (16.5) | -0.5 (20.47) | -4.08 (19.15) | -3.55 (24.45) | -3.00 (22.97) | -2.52 (16.71) |
|  | P-value |  | 0.045 | 0.198 | 0.033 | 0.767 | 0.131 | 0.134 | 0.674 | 0.16 |
| Future | n | 71 | 66 | 71 | 48 | 49 | 44 | 37 | 25 | 52 |
|  | Mean (SD)* | 38.94 (21.41) | -1.97 (12.31) | -3.17 (16.63) | -2.29 (16.14) | -0.1 (14.34) | -1.93 (15.56) | 1.35 (17.7) | 1.2 (15.29) | 1.15 (16.42) |
|  | P-value |  | 0.196 | 0.136 | 0.282 | 0.897 | 0.319 | 0.759 | 0.375 | 0.647 |
| Partnership and sexuality | n | 70 | 66 | 69 | 47 | 49 | 44 | 35 | 24 | 51 |
|  | Mean (SD)* | 14.76 (25.97) | 1.52 (19.22) | -0.48 (15.25) | -1.6 (15.11) | -0.34 (24.17) | -2.27 (21.14) | -5 (25.82) | -4.51 (18.87) | -0.49 (19.39) |
|  | P-value |  | 0.646 | 0.895 | 0.492 | 0.793 | 1 | 0.133 | 0.31 | 0.413 |
| Physical health | n | 72 | 66 | 68 | 47 | 49 | 44 | 36 | 24 | 51 |
|  | Mean (SD)* | 45.03 (24.28) | -13.67 (17.35) | -10.17 (20.14) | -12.05 (19.89) | -8.27 (25.88) | -5.85 (18.43) | -2.71 (23.48) | -7.97 (26.38) | -8.5 (20.02) |
|  | P-value |  | ≤ 0.01 | ≤ 0.01 | ≤ 0.01 | 0.047 | 0.016 | 0.468 | 0.044 | 0.004 |
| Sports and leisure | n | 59 | 50 | 54 | 38 | 39 | 31 | 27 | 20 | 40 |
|  | Mean (SD)* | 57.75 (23.25) | -10.1 (21.97) | -10.16 (24.05) | -11.15 (23.09) | -8.11 (26.45) | -14.48 (19.59) | -11.71 (31.22) | -10.62 (21.65) | -9.38 (25.59) |
|  | P-value |  | 0.003 | 0.003 | 0.007 | 0.053 | 0.002 | 0.177 | 0.182 | 0.004 |
| Treatment | n | 73 | 67 | 72 | 49 | 52 | 45 | 37 | 25 | 54 |
|  | Mean (SD)* | 31.08 (15.62) | -4.58 (15.85) | -2.76 (14.69) | -4.46 (13.47) | -5.39 (12.73) | -3.71 (14.66) | -0.88 (18.3) | -0.05 (15.21) | -2.4 (12.49) |
|  | P-value |  | 0.026 | 0.099 | 0.023 | 0.002 | 0.025 | 0.361 | 0.362 | 0.068 |
| View of yourself | n | 73 | 66 | 71 | 49 | 52 | 45 | 37 | 25 | 54 |
|  | Mean (SD)* | 41.16 (22.62) | -6.25 (15.12) | -5.42 (17.6) | -6.63 (16.34) | -2.07 (17.52) | -4.72 (13.65) | -1.89 (16.09) | -2.05 (18.11) | -3.43 (15.23) |
|  | P-value |  | 0.001 | 0.008 | 0.003 | 0.59 | 0.082 | 0.491 | 0.634 | 0.025 |
| Work and school | n | 63 | 54 | 58 | 38 | 39 | 35 | 29 | 21 | 45 |
|  | Mean (SD)* | 23.68 (23.04) | -1.81 (17.01) | -0.83 (19.42) | -2.25 (15.67) | 0.05 (20.10) | -4.88 (20.38) | -2.3 (17.62) | 0.69 (28.41) | 0.05 (17.41) |
|  | P-value |  | 0.305 | 0.542 | 0.404 | 0.736 | 0.143 | 0.603 | 0.439 | 0.575 |

*End of study could refer to any timepoint during B-LONG or B-YOND, depending on when the patient completed the study

P-value <0.05 indicates a statistically significant difference.

Haem-A-QoL, Haemophilia Quality of Life Questionnaire for Adults; SD, standard deviation.

**TABLE 3.** Haem-A-QoL item response for adults and adolescents reporting ‘never/rarely/seldom’ (originally enrolled in B-LONG).

| Item* | Patient population^†^ |  | Baseline  B-LONG | Month 6  B-LONG | Month 12  B-LONG | B-YOND  (VISIT 1) | Month 12/  B-YOND | Month 24/  B-YOND | Month 36/  B-YOND | Month 48/  B-YOND | End of study^‡^ |
| --- | --- | --- | --- | --- | --- | --- | --- | --- | --- | --- | --- |
| I didn't have the freedom to travel where I wanted | Previous treatment regimen prophylaxis | n | 37 | 30 | 38 | 34 | 35 | 28 | 21 | 11 | 35 |
|  |  | % ‘never/rarely/seldom’ | 64.86 | 76.67 | 63.16 | 67.65 | 68.57 | 71.43 | 71.43 | 81.82 | 88.57 |
|  |  | P-value |  | 2 | 1 | 1 | 1 | 1 | 0.683 | 1 | 0.131 |
|  | Previous treatment regimen on-demand | n | 30 | 31 | 30 | 22 | 27 | 24 | 20 | 17 | 26 |
|  |  | % ‘never/rarely/seldom’ | 63.33 | 61.29 | 76.67 | 86.36 | 70.37 | 70.83 | 70.00 | 94.12 | 65.38 |
|  |  | P-value |  | 0.564 | 0.221 | 0.134 | 0.724 | 0.724 | 1 | 0.248 | 0.617 |
|  | Total | n | 68 | 62 | 69 | 57 | 63 | 53 | 42 | 29 | 62 |
|  |  | % ‘never/rarely/seldom’ | 64.71 | 67.74 | 68.12 | 73.68 | 68.25 | 69.81 | 69.05 | 86.21 | 77.42 |
|  |  | P-value |  | 0.808 | 0.564 | 0.505 | 0.796 | 0.593 | 0.593 | 0.683 | 0.564 |
| I had difficulty walking as far as I wanted to | Previous treatment regimen prophylaxis | n | 38 | 32 | 38 | 34 | 34 | 32 | 22 | 11 | 34 |
|  |  | % ‘never/rarely/seldom’ | 36.84 | 71.88 | 57.89 | 58.82 | 47.06 | 53.12 | 40.91 | 54.55 | 52.94 |
|  |  | P-value |  | 0.004 | 0.043 | 0.07 | 0.546 | 0.131 | 1 | 1 | 0.752 |
|  | Previous treatment regimen on-demand | n | 33 | 33 | 30 | 22 | 27 | 25 | 22 | 20 | 31 |
|  |  | % ‘never/rarely/seldom’ | 39.39 | 66.67 | 66.67 | 63.64 | 59.26 | 52.00 | 63.64 | 55.00 | 54.84 |
|  |  | P-value |  | 0.027 | 0.046 | 0.131 | 0.343 | 0.289 | 0.131 | 0.221 | 0.505 |
|  | Total | n | 72 | 66 | 69 | 57 | 62 | 58 | 44 | 32 | 66 |
|  |  | % ‘never/rarely/seldom’ | 37.50 | 68.18 | 60.87 | 59.65 | 51.61 | 53.45 | 52.27 | 53.12 | 54.55 |
|  |  | P-value |  | ≤ 0.01 | 0.002 | ≤ 0.01 | 0.127 | 0.024 | 0.114 | 0.182 | 0.181 |
| I had pain in my joints | Previous treatment regimen prophylaxis | n | 39 | 34 | 39 | 34 | 34 | 32 | 22 | 11 | 34 |
|  |  | % ‘never/rarely/seldom’ | 23.08 | 50.00 | 41.03 | 38.24 | 38.24 | 37.50 | 27.27 | 45.45 | 38.24 |
|  |  | P-value |  | 0.013 | 0.131 | 0.371 | 0.48 | 0.617 | 1 | 1 | 1 |
|  | Previous treatment regimen on-demand | n | 33 | 33 | 31 | 23 | 28 | 25 | 22 | 20 | 31 |
|  |  | % ‘never/rarely/seldom’ | 27.27 | 48.48 | 48.39 | 52.17 | 60.71 | 44.00 | 54.55 | 45.00 | 58.06 |
|  |  | P-value |  | 0.046 | 0.077 | 0.131 | 0.046 | 0.371 | 0.221 | 0.617 | 0.289 |
|  | Total | n | 73 | 68 | 71 | 58 | 63 | 58 | 44 | 32 | 66 |
|  |  | % ‘never/rarely/seldom’ | 24.66 | 48.53 | 43.66 | 43.10 | 47.62 | 39.66 | 40.91 | 43.75 | 46.97 |
|  |  | P-value |  | 0.001 | ≤ 0.01 | 0.043 | 0.016 | 0.182 | 0.228 | 0.683 | 0.267 |
| I had to avoid sports like football | Previous treatment regimen prophylaxis | n | 31 | 23 | 31 | 28 | 24 | 23 | 15 | 8 | 27 |
|  |  | % ‘never/rarely/seldom’ | 16.13 | 43.48 | 25.81 | 28.57 | 29.17 | 26.09 | 33.33 | 12.50 | 40.74 |
|  |  | P-value |  | 0.041 | 0.074 | 0.074 | 0.248 | 1 | 0.074 | - | 0.074 |
|  | Previous treatment regimen on-demand | n | 24 | 23 | 25 | 17 | 20 | 21 | 17 | 17 | 24 |
|  |  | % ‘never/rarely/seldom’ | 4.17 | 17.39 | 36.00 | 52.94 | 25.00 | 28.57 | 52.94 | 35.29 | 37.5 |
|  |  | P-value |  | 0.248 | 0.023 | 0.023 | 0.248 | 0.134 | 0.041 | 0.134 | 0.134 |
|  | Total | n | 56 | 47 | 57 | 46 | 45 | 45 | 33 | 26 | 52 |
|  |  | % ‘never/rarely/seldom’ | 12.50 | 29.79 | 29.82 | 36.96 | 26.67 | 26.67 | 42.42 | 26.92 | 38.46 |
|  |  | P-value |  | 0.027 | 0.006 | 0.006 | 0.131 | 0.289 | 0.009 | 0.371 | 0.027 |
| I had to avoid sports that I like because of my hemophilia | Previous treatment regimen prophylaxis | n | 34 | 30 | 33 | 29 | 27 | 25 | 17 | 10 | 30 |
|  |  | % ‘never/rarely/seldom’ | 23.53 | 50.00 | 48.48 | 51.72 | 51.85 | 44.00 | 35.29 | 40.00 | 56.67 |
|  |  | P-value |  | 0.182 | 0.027 | 0.027 | 0.114 | 0.45 | 0.45 | 1 | 0.043 |
|  | Previous treatment regimen on-demand | n | 26 | 28 | 26 | 18 | 25 | 23 | 20 | 18 | 26 |
|  |  | % ‘never/rarely/seldom’ | 23.08 | 32.14 | 42.31 | 50.00 | 36.00 | 43.48 | 55.00 | 55.56 | 46.15 |
|  |  | P-value |  | 0.221 | 0.289 | 0.221 | 0.221 | 0.077 | 0.077 | 0.371 | 0.131 |
|  | Total | n | 61 | 59 | 60 | 48 | 53 | 49 | 38 | 29 | 57 |
|  |  | % ‘never/rarely/seldom’ | 22.95 | 40.68 | 45.00 | 50.00 | 43.40 | 42.86 | 44.74 | 41.38 | 50.88 |
|  |  | P-value |  | 0.039 | ≤ 0.01 | 0.006 | 0.024 | 0.039 | 0.039 | 0.289 | 0.006 |
| I played sports just as much as others | Previous treatment regimen prophylaxis | n | 33 | 25 | 31 | 28 | 25 | 23 | 16 | 9 | 28 |
|  |  | % ‘never/rarely/seldom’ | 66.67 | 52.00 | 54.84 | 57.14 | 40.00 | 56.52 | 43.75 | 44.44 | 42.86 |
|  |  | P-value |  | 0.221 | 0.683 | 1 | 0.131 | 1 | 0.371 | 0.48 | 0.041 |
|  | Previous treatment regimen on-demand | n | 27 | 29 | 26 | 18 | 23 | 19 | 18 | 17 | 27 |
|  |  | % ‘never/rarely/seldom’ | 59.26 | 48.28 | 34.62 | 16.67 | 34.78 | 42.11 | 38.89 | 29.41 | 37.04 |
|  |  | P-value |  | 0.134 | 0.077 | 0.041 | 0.131 | 0.074 | 0.131 | 0.131 | 0.221 |
|  | Total | n | 61 | 55 | 58 | 47 | 49 | 43 | 34 | 27 | 56 |
|  |  | % ‘never/rarely/seldom’ | 62.30 | 49.09 | 44.83 | 40.43 | 36.73 | 48.84 | 41.18 | 33.33 | 39.29 |
|  |  | P-value |  | 0.027 | 0.061 | 0.07 | 0.016 | 0.114 | 0.043 | 0.046 | 0.009 |
| I was annoyed about the amount of time spent having the injections | Previous treatment regimen prophylaxis | n | 39 | 33 | 39 | 34 | 35 | 31 | 22 | 11 | 36 |
|  |  | % ‘never/rarely/seldom’ | 74.36 | 84.85 | 89.74 | 91.18 | 88.57 | 83.87 | 81.82 | 72.73 | 88.89 |
|  |  | P-value |  | 0.546 | 0.228 | 0.182 | 0.182 | 0.683 | 0.617 | - | 0.289 |
|  | Previous treatment regimen on-demand | n | 33 | 33 | 32 | 23 | 28 | 25 | 22 | 20 | 31 |
|  |  | % ‘never/rarely/seldom’ | 63.64 | 81.82 | 75.00 | 73.91 | 82.14 | 88.00 | 77.27 | 75.00 | 74.19 |
|  |  | P-value |  | 0.149 | 0.343 | 0.221 | 0.074 | 0.074 | 1 | 1 | 0.134 |
|  | Total | n | 73 | 67 | 72 | 58 | 64 | 57 | 44 | 32 | 68 |
|  |  | % ‘never/rarely/seldom’ | 68.49 | 82.09 | 81.94 | 82.76 | 84.38 | 84.21 | 79.55 | 71.88 | 80.88 |
|  |  | P-value |  | 0.061 | 0.05 | 0.039 | 0.016 | 0.070 | 0.724 | 1 | 0.043 |
| It was necessary for me to plan everything in advance | Previous treatment regimen prophylaxis | n | 38 | 32 | 39 | 34 | 35 | 29 | 21 | 11 | 35 |
|  |  | % ‘never/rarely/seldom’ | 34.21 | 59.38 | 41.03 | 44.12 | 45.71 | 55.17 | 38.10 | 45.45 | 62.86 |
|  |  | P-value |  | 0.07 | 0.752 | 0.505 | 0.546 | 0.228 | 1 | 1 | 0.016 |
|  | Previous treatment regimen on-demand | n | 31 | 31 | 31 | 22 | 27 | 24 | 18 | 17 | 29 |
|  |  | % ‘never/rarely/seldom’ | 19.35 | 35.48 | 41.94 | 54.55 | 37.04 | 41.67 | 38.89 | 47.06 | 48.28 |
|  |  | P-value |  | 0.228 | 0.114 | 0.114 | 0.343 | 0.182 | 0.45 | 0.134 | 0.228 |
|  | Total | n | 70 | 64 | 71 | 57 | 63 | 54 | 40 | 29 | 65 |
|  |  | % ‘never/rarely/seldom’ | 27.14 | 46.88 | 40.85 | 47.37 | 41.27 | 48.15 | 37.50 | 44.83 | 55.38 |
|  |  | P-value |  | 0.011 | 0.074 | 0.039 | 0.127 | 0.025 | 0.405 | 0.450 | 0.006 |
| It was painful for me to move | Previous treatment regimen prophylaxis | n | 38 | 33 | 38 | 34 | 33 | 32 | 21 | 11 | 34 |
|  |  | % ‘never/rarely/seldom’ | 44.74 | 51.52 | 57.89 | 55.88 | 60.61 | 50.00 | 47.62 | 63.64 | 55.88 |
|  |  | P-value |  | 0.724 | 0.343 | 0.505 | 0.343 | 1 | 0.683 | 0.48 | 0.45 |
|  | Previous treatment regimen on-demand | n | 33 | 33 | 30 | 22 | 27 | 25 | 21 | 20 | 30 |
|  |  | % ‘never/rarely/seldom’ | 33.33 | 57.58 | 53.33 | 63.64 | 62.96 | 52.00 | 47.62 | 75.00 | 46.67 |
|  |  | P-value |  | 0.043 | 0.114 | 0.046 | 0.149 | 0.343 | 1 | 0.077 | 0.683 |
|  | Total | n | 72 | 67 | 69 | 57 | 61 | 57 | 42 | 32 | 65 |
|  |  | % ‘never/rarely/seldom’ | 38.89 | 53.73 | 55.07 | 57.89 | 60.66 | 50.88 | 47.62 | 71.88 | 52.31 |
|  |  | P-value |  | 0.025 | 0.025 | 0.034 | 0.033 | 0.346 | 0.763 | 0.228 | 0.181 |
| My swellings hurt | Previous treatment regimen prophylaxis | n | 38 | 32 | 37 | 33 | 34 | 32 | 22 | 11 | 33 |
|  |  | % ‘never/rarely/seldom’ | 39.47 | 62.50 | 59.46 | 57.58 | 58.82 | 59.38 | 45.45 | 45.45 | 54.55 |
|  |  | P-value |  | 0.228 | 0.149 | 0.505 | 0.343 | 0.546 | 0.683 | 0.248 | 1 |
|  | Previous treatment regimen on-demand | n | 31 | 33 | 31 | 22 | 28 | 25 | 21 | 19 | 31 |
|  |  | % ‘never/rarely/seldom’ | 35.48 | 66.67 | 74.19 | 68.18 | 75.00 | 68.00 | 66.67 | 68.42 | 67.74 |
|  |  | P-value |  | 0.027 | 0.006 | 0.041 | 0.027 | 0.077 | 0.45 | 0.131 | 0.505 |
|  | Total | n | 70 | 65 | 69 | 56 | 63 | 57 | 44 | 31 | 65 |
|  |  | % ‘never/rarely/seldom’ | 38.57 | 64.62 | 65.22 | 66.07 | 65.08 | 63.16 | 54.55 | 58.06 | 61.54 |
|  |  | P-value |  | 0.004 | 0.002 | 0.016 | 0.016 | 0.039 | 1 | 0.763 | 0.491 |

*Sum of patients for whom results are shown by previous treatment regimen may differ by 1 from the total for the two subgroups due to missing data for one patient; ^†^Treatment regimen prior to enrolling in B-LONG; ^‡^End of study could refer to any timepoint during B-LONG or B-YOND, depending on when the patient completed the study.

Haem-A-QoL, Haemophilia Quality of Life Questionnaire for Adults; SD, standard deviation
